# Maximizing the Radiation Use Efficiency by Matching the Leaf Area and Leaf Nitrogen Vertical Distributions in a Maize Canopy: A Simulation Study

**DOI:** 10.34133/plantphenomics.0217

**Published:** 2024-07-29

**Authors:** Baiyan Wang, Shenghao Gu, Junhao Wang, Bo Chen, Weiliang Wen, Xinyu Guo, Chunjiang Zhao

**Affiliations:** ^1^ Beijing Key Lab of Digital Plant, Information Technology Research Center, Beijing Academy of Agriculture and Forestry Sciences, Beijing 100097, China.; ^2^ Nanjing Agricultural University, MSU Institute, Nanjing 210095, China.; ^3^ China Agricultural University, College of Resources and Environmental Sciences, Beijing 100193, China.

## Abstract

The radiation use efficiency (RUE) is one of the most important functional traits determining crop productivity. The coordination of the vertical distribution of light and leaf nitrogen has been proven to be effective in boosting the RUE from both experimental and computational evidence. However, previous simulation studies have primarily assumed that the leaf area is uniformly distributed along the canopy depth, rarely considering the optimization of the leaf area distribution, especially for C4 crops. The present study hypothesizes that the RUE may be maximized by matching the leaf area and leaf nitrogen vertical distributions in the canopy. To test this hypothesis, various virtual maize canopies were generated by combining the leaf inclination angle, vertical leaf area distribution, and vertical leaf nitrogen distribution and were further evaluated by an improved multilayer canopy photosynthesis model. We found that a greater fraction of leaf nitrogen is preferentially allocated to canopy layers with greater leaf areas to maximize the RUE. The coordination of light and nitrogen emerged as a property from the simulations to maximize the RUE in most scenarios, particularly in dense canopies. This study not only facilitates explicit and precise profiling of ideotypes for maximizing the RUE but also represents a primary step toward high-throughput phenotyping and screening of the RUE for massive numbers of inbred lines and cultivars.

## Introduction

Given the persistent challenges posed by population growth and climate change, ensuring food security has become an urgent concern for the agricultural sector [1,2]. Maize, the world’s most widely produced and productive grain crop, plays a vital role in ensuring global food security [3,4]. However, the radiation use efficiency (RUE) of crops, which refers to the efficiency of converting intercepted radiation into dry matter, has been estimated to be less than one-third of its theoretical maximum value [5,6]. This indicates that there is substantial potential for improving crop yield. The RUE is widely recognized as one of the major determinants of crop productivity and can be estimated by multiplying the RUE by the intercepted radiation and the harvest index during the crop growth period [7]. It is worth noting that once the canopy of maize closes, it effectively intercepts almost all incident radiation [8]. In addition, the harvest index of maize in most major growing areas has remained stable over the past decades [9]. Therefore, breeding maize with a relatively high RUE is an essential and promising approach for achieving high yields.

The spatial distributions of light and leaf nitrogen content are 2 significant factors influencing the RUE [10,11]. Light availability and leaf nitrogen content per unit leaf area [specific leaf nitrogen (SLN)] exhibit a vertically decreasing pattern toward the canopy bottom with increasing cumulative leaf area index (CLAI) [12]. This pattern is quantitatively described using the extinction coefficient of light (*K*_L_) [13] and SLN (*K*_N_) [14] together with the canopy leaf area index (LAI) [15]. In recent decades, the release of maize cultivars has led to a decreasing trend in *K*_L_ worldwide. This is primarily attributed to modifications in leaf inclination angles (LIAs) and, to some extent, changes in the leaf area distribution [16–18]. Apart from light conditions, the SLN distribution within the canopy influences canopy photosynthesis, as it is positively associated with the leaf photosynthetic capacity [19,20]. Numerous studies based on optimization theory and the large-leaf scaling method have concluded that the SLN should be distributed in proportion to local irradiance to maximize canopy photosynthesis [14,21,22]. The light and leaf nitrogen in the canopy are optimally distributed when the *K*_N_/*K*_L_ ratio equals 1 and suboptimally distributed when the ratio deviates from 1 [23–25]. Actual light-SLN profiles in canopies have been shown to be suboptimal in crops [25,26], indicating the potential to further increase canopy photosynthesis by approximately 20% [23,27].

Genetic breeding techniques have increasingly been utilized to select for secondary traits that contribute to the RUE [28]. Genome-wide association analyses have been widely used to identify quantitative trait loci associated with the LIA [29], leaf area [30], and nitrogen metabolism [31]. Progress has been made to develop more compact maize strains with larger LIAs [30], optimize the canopy structure with an appropriate leaf area distribution [18], and enhance the photosynthetic efficiency through improved nitrogen partitioning strategies [32]. As a result, it is feasible to quantitatively characterize leaf angle, leaf area vertical distribution, and nitrogen allocation that align with the optimal distribution of light and nitrogen when constructing the ideal maize ideotype. However, quantifying the contributions of different combinations of secondary traits, such as yield and RUE, to breeding targets requires crop simulation tools [33,34].

Canopy photosynthesis models have evolved continuously to predict optimal light and leaf nitrogen profiles [14,21,22,25,35]. Field [21] initially discovered that canopy photosynthesis is maximized when leaf nitrogen is distributed in a way that maintains identical marginal carbon gains from nitrogen investment for each layer across the canopy. This theory has subsequently been supported by simulation studies focused on optimizing canopy photosynthesis and specified as the matching between SLN and local irradiance [14,22,36]. Furthermore, 3-dimensional canopy photosynthesis was developed to optimize canopy architectural traits (e.g., leaf width, stem height, and leaf angle) and maximize canopy photosynthetic production in rice [37]. While considerable attention has been given to optimizing the canopy structure in C3 crops, little attention has been given to optimizing the canopy structure in C4 crops. In a simulation study using a multilayer canopy photosynthesis model, Bonelli and Andrade [35] examined the optimal distribution of SLN within a canopy to maximize the RUE and concluded that the optimal pattern largely depends on the LAI and nitrogen availability. However, they did not consider the effect of LIA and assumed that the leaf area is distributed uniformly in the model, which contradicts the fact that the leaf area is often unevenly distributed along the canopy [38]. Neglecting the leaf area distribution in simplified models can lead to discrepancies, as it significantly affects simulated energy fluxes [39]. However, no analysis has evaluated its role in determining the canopy RUE to date. According to [11], we hypothesize that the RUE may be maximized by matching the vertical distributions of both the leaf area and leaf nitrogen in the canopy.

The objectives of this study are threefold: (a) to test whether the RUE can be maximized by matching the vertical distributions of both the leaf area and leaf nitrogen; (b) to quantitatively characterize the LIA, LAI, and vertical distribution of leaf area and leaf nitrogen in the maize canopy to maximize the RUE; and (c) to determine to what extent the emergent optimal light-SLN pattern from our simulations deviates from that based on optimization theory. Ultimately, this study aimed to identify the maize ideotype that exhibits high photosynthetic efficiency and to provide complementary evidence to optimize canopy photosynthesis theory.

## Materials and Methods

### Canopy photosynthesis model

A canopy photosynthesis model based on the multilayer model was used [35] to simulate canopy photosynthetic production. The model framework consisted of 3 major modules: light distribution, canopy photosynthesis, and dry mass production (Table S1). In the module of light distribution, we included the LIA distribution model instead of the spherical distribution model to optimize the LIA and included a vertical distribution model of the leaf area instead of an evenly distributed leaf area pattern. In the canopy photosynthesis module, we considered the vertical distribution of the leaf nitrogen content in the canopy. These functions were integrated into the multilayer canopy photosynthesis model to optimize the canopy structural and functional traits and to maximize the RUE.

#### Light distribution module

The light interception of the canopy is influenced by the leaf angle and leaf area. By simulating the frequency distribution of the LIA and the vertical distribution of the leaf area, we included the LIA distribution model instead of the spherical distribution model to optimize the canopy structure. Each leaf layer is further partitioned into a sunlit part that receives both direct and diffuse light and a shaded part that receives only scattered light to simulate the light interception characteristics of the canopy.

The extinction coefficient of direct photosynthetically active radiation (PAR) [*K*_dir(*t*)_] at time *t* was calculated as follows:

$$K_{\text{dir}\left(t\right)}=\frac{O_{\text{av}}}{\text{sin}\beta_{\left(t\right)}}$$ (1)

where *β*_(*t*)_ is the solar altitude angle at time *t* of the day (in degrees) and *O*_av_ is the average projection of the leaves in the direction of the solar beam. On the basis of the hypothesis that leaves in the canopy have a uniform azimuthal orientation, *O*_av_ can be calculated as follows:

Oava=sinβtcosβLa2π[sinβtcosβLaarcsintanβttanβLa+sin2βLa−sin2βt],βt≥βLa,βt<βLa (2)

$$O_{\mathrm{av}}=\sum_{a=1}^{9}f_{a}O_{\mathrm{av}\left(a\right)}$$ (3)

where *β*_*L*(*a*)_, *f_a_*, and *O*_av(*a*)_ indicate the LIA (in degrees), the frequency of leaves, and the solar projection at the *a*th LIA class, respectively.

The 2-parameter beta distribution function [40] has been proven to be the most robust and appropriate distribution for describing the leaf inclination probability density [*f*(*b*)] [41] and is capable of reproducing 6 common theoretical leaf inclination distributions [42]. Moreover, *f_a_* is calculated as the integral of *f*(*b*) in a specific LIA class as follows:

$$f_{a}=\int_{a}^{a+1}f\left(b\right)$$ (4)

$$f\left(b\right)=\frac{1}{B(\mu ,\nu )}{\left(1-b\right)}^{\mu -1}b^{\nu -1}$$ (5)

where *b* = 2*β*_*L*(*a*)_/*π* and *β*_*L*(*a*)_ is expressed in radians. The beta distribution *B*(*μ*,*ν*) is defined as follows:

$$B\left(\mu ,\nu \right)=\frac{\Gamma \left(\mu \right)\Gamma \left(\nu \right)}{\Gamma \left(\mu +\nu \right)}$$ (6)

where Γ is the gamma function and *μ* and *ν* are 2 parameters of the beta distribution.

The direct PAR [*I*_dir(*n,t*)_; in micromoles of photons per square meter per second] and diffuse PAR [*I*_diff(*n,t*)_; in micromoles of photons per square meter per second] per unit ground area at the top of the *n*th canopy layer at hour *t* of the day were calculated as follows:

$$I_{\text{dir}\left(n,t\right)}=I_{\text{dir}\left(0,t\right)}e^{-K_{\text{dir}\left(t\right)}\;{\text{CLAI}}_{n}}$$ (7)

$$I_{\text{diff}\left(n,t\right)}=I_{\text{dif}\left(0,t\right)}e^{-K_{\text{diff}}\;{\text{CLAI}}_{n}}$$ (8)

where *K*_diff_ is the PAR extinction coefficient for diffuse radiation, which was set as 0.7 [35]; *I*_dir(0,*t*)_ (in micromoles of photons per square meter per second) and *I*_diff(0,*t*)_ (in micromoles of photons per square meter per second) are the direct and diffuse incident PAR, respectively, at hour *t* of the day; and CLAI*_n_* is the cumulative leaf area index at the top of the *n*th canopy layer.

The vertical distribution of the leaf area as a function of the canopy depth in maize follows a bell shape, in which the LAI of an individual layer reaches the maximum at a certain layer [36]. The relationship between the CLAI*_n_* and canopy depth (*Z_n_*) was accordingly quantified using a beta function [43] as follows:

$${\text{CLAI}}_{n}=\text{LAI}\left(1+\frac{Z_{e}-Z_{n}}{Z_{e}-Z_{m}}\right){\left(\frac{Z_{n}}{Z_{e}}\right)}^{\frac{Z_{e}}{Z_{e}-Z_{m}}}$$ (9)

where *Z_e_* is the total canopy depth of maize, *Z_m_* is the canopy depth at a given canopy interval where the corresponding LAI reaches the maximum, and *Z_n_* is the canopy depth at the bottom of the *n*th canopy layer that is measured from the top downward. *Z_n_* is equal to 0 at the canopy top and to the canopy height at the canopy bottom. To simplify the canopy structure and improve the computational efficiency, the canopy was divided into 10 equally spaced layers. The leaf area profile (LAP*_n_*) was defined as the leaf area per unit of land area of the *n*th individual canopy layer [44] and was calculated as the difference between CLAI*_n_* and CLAI_*n−*1_.

The PAR intercepted by the entire canopy during the day [*I*_PAR(DAY)_; in megajoules per square meter per day] was calculated as follows:

$$\Delta I_{\text{dir}\left(n,t\right)}=I_{\text{dir(}\text{n,t}\text{)}}-I_{\text{dir}\text{(n+1,t}\text{)}}$$ (10)

$$\Delta I_{\text{diff}\left(n,t\right)}=I_{\text{diff(}\text{n,t}\text{)}}-I_{\text{diff}\left(n+1,t\right)}$$ (11)

$$I_{\text{PAR}\left(t\right)}=\sum_{n=1}^{10}\left(\Delta I_{\text{dir}\left(n,t\right)}+\Delta I_{\text{diff}\left(n,t\right)}\right)$$ (12)

$$I_{\text{PAR}\left(\text{DAY}\right)}=\frac{3,600\sum_{t=\text{sunrise}}^{\text{sunset}}I_{\text{PAR}\left(t\right)}}{4.55}10^{-6}$$ (13)

where a value of 4.55 is used for the conversion of micromoles per square meter per second to joules per square meter per second [45]. During the postsilking stage of maize in Beijing, the sunrise and sunset times are approximately 6 AM and 6 PM, respectively.

#### Canopy photosynthesis module

The photosynthesis rate was first calculated for sunlit [*A*_sun(*n,a,t*)_] and shaded leaves [*A*_sh(*n,t*)_] separately, and they were integrated by accounting for the distribution of the LIA (*f_a_*) and the proportion of positive leaves [*f*_sun(*n,t*)_] across canopy layers to simulate the instantaneous canopy photosynthesis rate [*A*_can(*t*)_]. Finally, the daily assimilation of CO_2_ in the canopy [*A*_can(DAY)_] was obtained by integrating *A*_can(*t*)_ at the daily scale.

The fraction of sunlit [*f*_sun(*n,t*)_] and shaded [*f*_sh(*n,t*)_] leaves in the *n*th canopy at hour *t* of the day was calculated as follows:

$$f_{\mathrm{sun}\left(n,t\right)}=e^{-K_{\text{dir}\left(t\right)}\frac{{\text{CLAI}}_{n}+{\text{CLAI}}_{n+1}}{2}}$$ (14)

$$f_{\mathrm{sh}\left(n,t\right)}=1-f_{\text{sun}\left(n,t\right)}$$ (15)

where CLAI*_n_* and CLAI_*n*+1_ indicate the cumulative leaf area index at the *n*th and (*n* + 1)th canopy layers, respectively.

The instantaneous photosynthetic rates of sunlit leaves [*A*_sun(*n,t*)_] and shaded leaves [*A*_sh(*n,t*)_] at time *t* of the *n*th canopy of the day were calculated using a nonrectangular hyperbolic function as follows:

$$A_{\text{sun}\left(n,a,t\right)}=\frac{\alpha I_{\text{sun}\left(n,a,t\right)}+A_{\text{max}\left(n\right)}-\sqrt{{\left(\alpha I_{\text{sun}\left(n,a,t\right)}+A_{\text{max}\left(n\right)}\right)}^{2}-4\theta \alpha I_{\text{sun}\left(n,a,t\right)}A_{\text{max}\left(n\right)}}}{2\theta }$$ (16)

$$A_{\mathrm{sh}\left(n,t\right)}=\frac{\alpha I_{\mathrm{sh}\left(n,t\right)}+A_{\text{max}\left(n\right)}-\sqrt{{\left(\alpha I_{\mathrm{sh}\left(n,t\right)}+A_{\text{max}\left(n\right)}\right)}^{2}-4\theta \alpha I_{\mathrm{sh}\left(n,t\right)}A_{\text{max}\left(n\right)}}}{2\theta }$$ (17)

$$I_{\text{sun}\left(n,a,t\right)}=I_{\text{dir}\left(0,t\right)}\frac{\text{cos}\zeta_{\left(a,t\right)}}{\text{sin}\beta_{\left(t\right)}}+I_{sh\left(n,t\right)}$$ (18)

$$I_{\mathrm{sh}\left(n,t\right)}=\frac{\Delta I_{\text{diff}\left(n,t\right)}}{{\mathrm{LAP}}_{n}}$$ (19)

where *α* is the apparent quantum efficiency with a value of 0.05, *θ* is an empirical coefficient of 0.8, *I*_sun(*n,a,t*)_ and *I*_sh(*n,t*)_ are the intercepted PAR per unit leaf area of the sunlit and shaded leaves of the *n*th canopy of the day at time *t*, respectively, and *ζ*_(*a*,*t*)_ indicates the LIA relative to the direct radiation.

*A*_max(*n*)_ indicates the maximum light-saturated leaf CO_2_ assimilation rate (in micromoles of CO_2_ per square meter per second) at the *n*th canopy, calculated from the relationship between the SLN and the photosynthetic rate (*A*_max_) [35] as follows:

$$A_{\text{max}\left(n\right)}=\frac{7.359\times \left(\frac{2}{1+e^{-4.724\times \left({\text{SLN}}_{n}-0.25\right)}}-1\right)\times 10^{-6}}{44\times 3600}$$ (20)

$${\text{LA}}_{n}=\frac{{\text{LAP}}_{n}}{\rho }$$ (21)

$${\text{SLN}}_{n}=\frac{{\text{LNC}}_{n}}{{\mathrm{LA}}_{n}}$$ (22)

where SLN*_n_* is the specific leaf nitrogen (in grams per square meter) in the *n*th canopy, LNC*_n_* is the leaf nitrogen content in the *n*th canopy, and *ρ* is the planting density, which has a value of 6.5 plants·m^−2^.

The instantaneous photosynthetic rate [*A*_can(*t*)_] for the entire canopy at time *t* of the day was calculated as follows:

$$A_{\mathrm{can}\left(t\right)}=\sum_{n=1}^{10}{\text{LAP}}_{n}\left\{\left[f_{\text{sun}\left(n,t\right)}\left(\sum_{a=1}^{9}f_{a}A_{\text{sun}\left(n,a,t\right)}\right)\right]+f_{\mathrm{sh}\left(n,t\right)}A_{\mathrm{sh}\left(n,t\right)}\right\}$$ (23)

The daily canopy assimilation of CO_2_ [*A*_can(DAY)_] was obtained by integrating *A*_can(*t*)_ to the daily scale via the following equation:

$$A_{\text{can}\left(\text{DAY}\right)}=3,600\sum_{t=\text{sunrise}}^{\text{sunset}}A_{\mathrm{can}\left(t\right)}$$ (24)

#### Dry matter accumulation module

The daily canopy accumulation of aboveground dry matter [DM_(DAY)_; in grams per square meter per day] was calculated as follows [46]:

$$DM_{\left(\text{DAY}\right)}=44A_{\mathrm{can}\left(\text{DAY}\right)}B\times 10^{-6}$$ (25)

where 44 is the molar mass of CO_2_ (in grams per mole) and *B* is the dry matter produced per unit mass of CO_2_ (0.41 for maize according to the results of [10]). The accumulated dry matter (ADM; in grams per square meter) over the postsilking period was calculated by the sum of DM_(DAY)_.

The model exported DM_(DAY)_ and *I*_PAR(DAY)_ in a daily step during the postsilking period. The RUE (in grams per megajoule) of the maize canopy was calculated by the slope of the linear relationship between the accumulated DM_(DAY)_ and *I*_PAR(DAY)_ over the entire postsilking period_._

### Simulation scenarios and environmental input

Leaf inclination distributions (*n* = 28) were combined with leaf nitrogen distributions (*n* = 28) to generate 784 scenarios, each of which was further integrated into 25 virtual canopy structures consisting of 5 LAIs, each with 5 different vertical distribution patterns of the leaf area. A total of 19,600 scenarios were generated to cover a wide range of canopy structural and functional traits. The average hourly incoming direct and diffuse PAR intensities from DOY(day of year) 200 to DOY 260 (i.e., postsilking period) during 2011–2020 in Tongzhou District, Beijing (39°56′ N, 116°41′ E) were set as the environmental inputs. The selected period was consistent with the maize postsilking period, during which the LAI reached its maximum and dry matter accumulation played a critical role in yield formation. Hourly direct and diffuse irradiance data were acquired from the Beijing Meteorological Service. All simulations were performed at a density of 6.5 plants·m^−2^.

#### Leaf inclination distribution scenarios

**Fig. 1.**
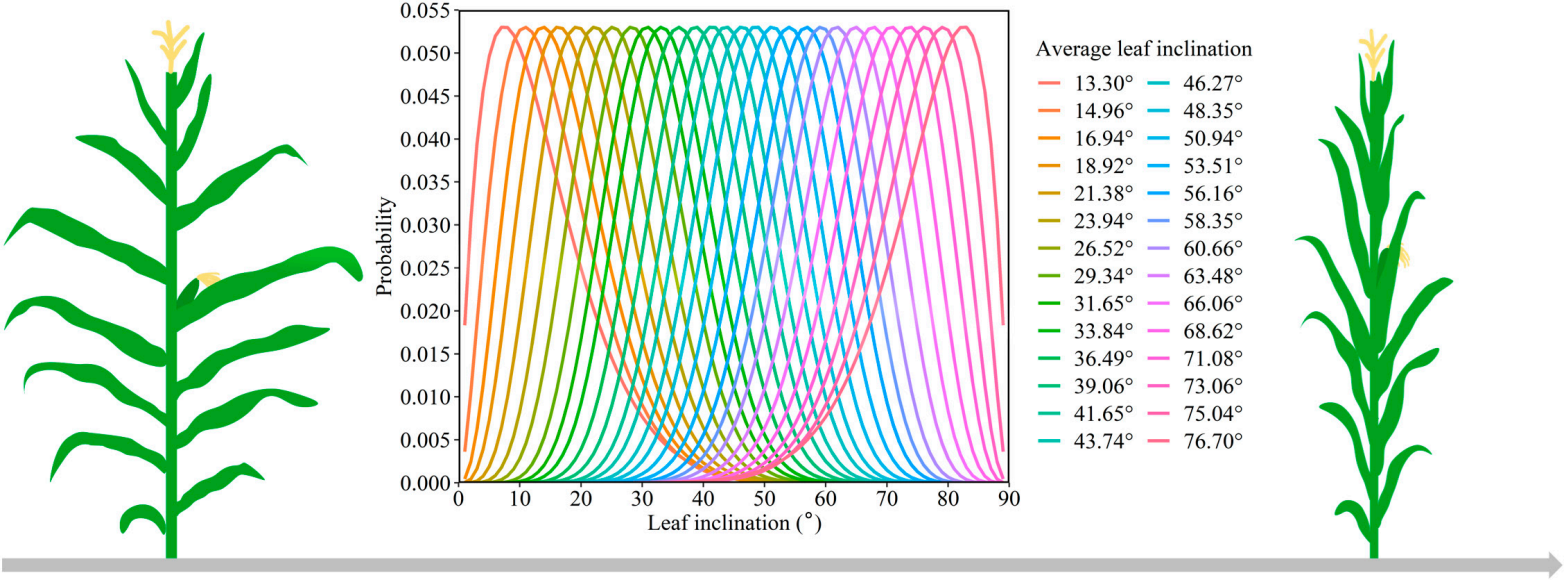
Leaf inclination distributions simulated using a beta distribution function. Different colors represent different leaf inclination distributions with different average values ranging from 13.30° to 76.70°, covering maize plants with horizontal leaves to erect leaves.

The beta distribution function was used to generate 28 canopy structures with different average LIAs ranging from 13.30° to 76.70° with similar peaks by modifying 2 parameters, *μ* and *ν,* using the “trial and error” method. Different canopy layers were assumed to follow the same leaf inclination distribution. The *f_a_* value was calculated by integrating the probability of each leaf inclination class. The leaf inclination distribution (Fig. 1) and probability of 9 inclination classes (Table S2) are listed in the Supplementary Materials.

#### Leaf nitrogen distribution scenarios

**Fig. 2.**
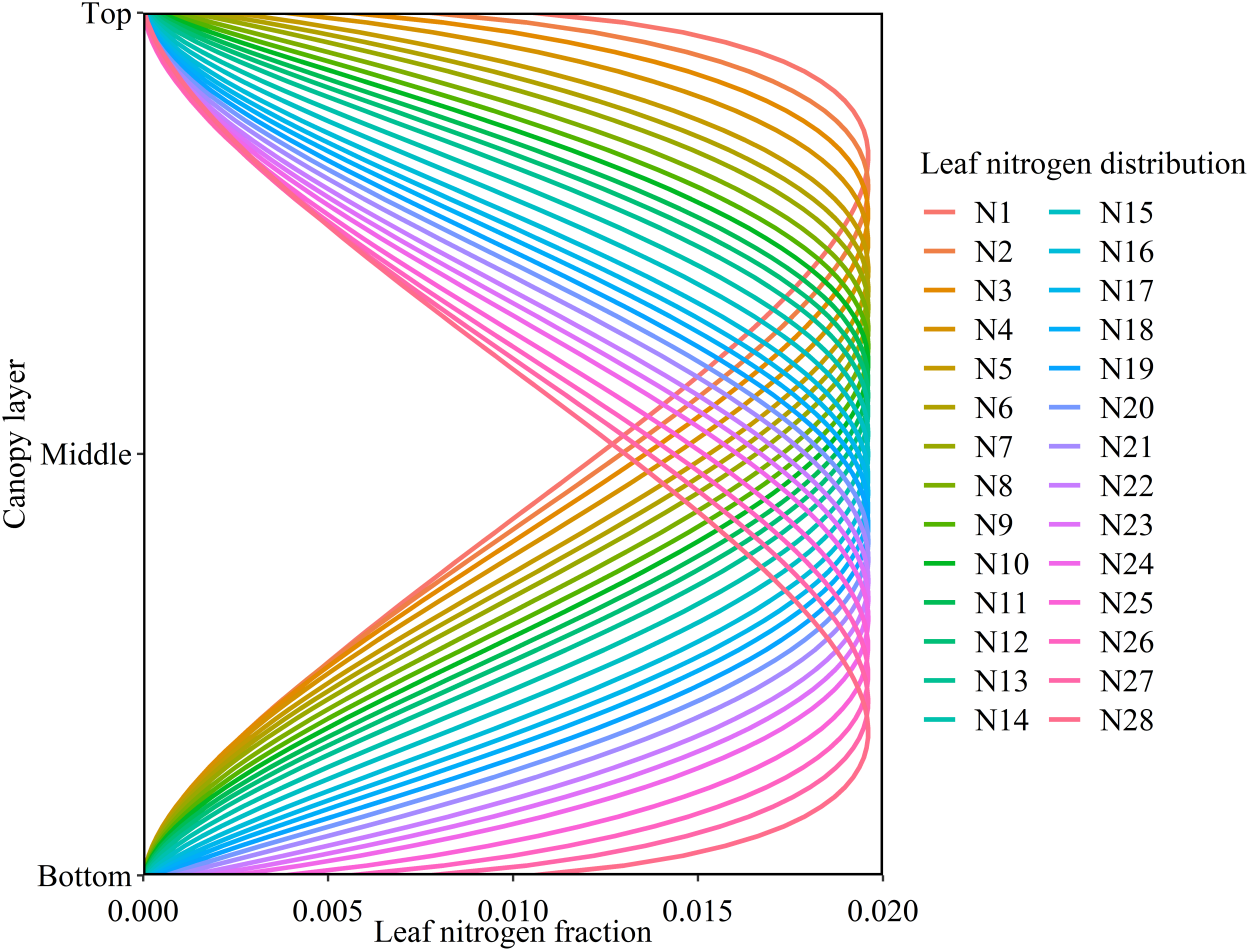
Different vertical distributions of the leaf nitrogen fraction simulated using a beta distribution function.

The vertical distribution of the leaf nitrogen content among 12 different groups of maize canopies was obtained from the literature [47–49]. Published data were extracted by Engauge digitization software (http://digitizer.sourceforge.net). The vertical distribution of canopy nitrogen content data was evaluated using the “fitdistrplus” package in the R programming language. Specifically, the “descdist” and “fitdist” functions were utilized to evaluate the distributions of the collected data. The results indicated that the distributions yielded by the “descdist” function were reasonably close to the exponential, gamma, and beta distributions. Finally, the “fitdist” function was applied to determine the degree of fit of the data to these 3 distributions [50]. The results showed that the vertical distributions of the canopy foliar nitrogen content mostly followed a beta distribution function (Table 1). The total leaf nitrogen content of an individual plant (LNC_tot_) was fixed at 900 mg·m^−2^ for each virtual canopy. The LNC*_n_* value was calculated by multiplying LNC_tot_ by the proportion of leaf nitrogen for the *n*th canopy layer generated from the following distribution. The leaf photosynthetic capacity was calculated by Eq. 20. Then, the beta distribution function was used to generate 28 different leaf nitrogen distributions with similar peaks but at different layers by modifying 2 parameters, *μ* and *ν,* using the “trial and error” method. The leaf nitrogen fraction of each canopy layer was calculated by integrating the fraction in each specific layer. The fraction of leaf nitrogen for a specific canopy layer, expressed by the ratio of the leaf nitrogen content to the total leaf nitrogen in a canopy, was utilized to represent the leaf nitrogen allocation strategy. The leaf nitrogen distribution is presented in Fig. 2, and the corresponding fraction and content values are listed in Table S3.

**Table 1.** Fitting test of the gamma, exponential, and beta distributions to the leaf nitrogen fraction in the different canopy layers.

Data group	Data source	Standard error		
		Gamma	Exponential	Beta
Group 1	[47]	1.213	3.162	1.127
Group 2		1.434	2.450	1.239
Group 3		1.691	2.450	1.462
Group 4		1.717	2.450	1.489
Group 5		1.922	2.450	1.655
Group 6	[48]	2.444	3.000	2.210
Group 7		2.828	3.000	2.492
Group 8	[49]	1.049	3.162	0.970
Group 9		1.469	3.162	1.352
Group 10		2.550	3.162	2.343
Group 11		3.533	3.000	3.201
Group 12		2.877	3.162	2.653

#### LAI and its vertical pattern

**Fig. 3.**
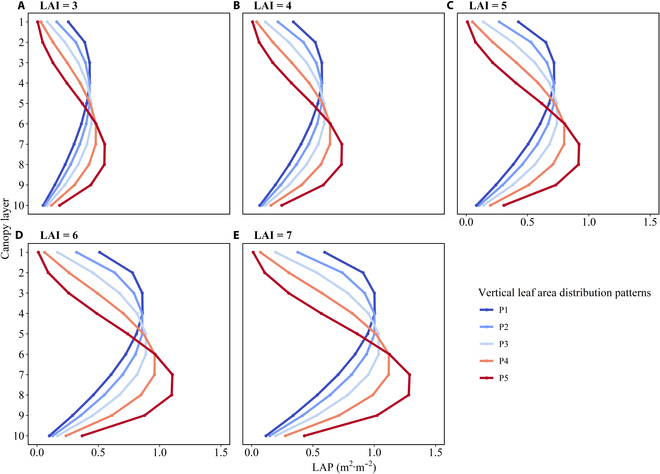
Different vertical distributions of the leaf area in the maize canopy for different LAIs ranging from 3 to 7 [(A) for 3, (B) for 4, (C) for 5, (D) for 6, and (E) for 7]. Different colors represent different vertical leaf area distribution patterns.

Twenty-five maize canopies with different plant leaf areas and vertical distributions of the leaf area were generated by modifying the LAI and *Z*_*m*_ (Eq. 9), respectively. The LAI was set to 3, 4, 5, 6, and 7 (Fig. 3). For each LAI, *Z_m_* was set to 0.3*Z_e_*, 0.4*Z_e_*, 0.5*Z_e_*, 0.6Z*_e_*, and 0.7*Z_e_* to generate canopies with different vertical leaf area distribution patterns, in which the peak of the beta-shaped pattern shifted from the upper to the lower canopy (Fig. 3).

### Comparison with the optimal light-SLN distribution via optimization theory

To examine the vertical distribution of light and the SLN, the IPAR_(*n*,*t*)_ value at 12:00 on a clear day was collected from the simulations. The relationship between the penetrated PAR and the SLN was described by a power function [51]:

$$\frac{{\text{SLN}}_{n}}{{\text{SLN}}_{0}}={\left(\frac{{\text{IPAR}}_{n}}{{\text{IPAR}}_{0}}\right)}^{K_{N}/K_{L}}$$ (26)

where *SLN*_0_ and SLN*_n_* are the specific leaf nitrogen at the top layer and the *n*th layer counting from the top, IPAR_0_ and IPAR*_n_* are the PAR values above the canopy top and at layer *n*, and *K*_N_ and *K*_L_ are the extinction coefficients of nitrogen and light, respectively. Canopy photosynthesis was regarded to be maximized by vertically matching the leaf nitrogen distribution and light distribution in a dense canopy, i.e., the *K*_N_/*K*_L_ ratio is equal to 1 according to optimization theory [22,23].

### Validation of the optimal leaf nitrogen partitioning pattern and leaf area distribution

To validate the optimal leaf area and leaf nitrogen distributions, corresponding measurements from the modern erect cultivar DH618 with the maximal yield recorded from 2013 to 2015 in China and the second-highest RUE in 2017 were obtained from published literature (LAI from Liu et al. [52] and N proportion from Liu et al. [48]). To ensure consistency, we selected the scenario with the maximum RUE at an LAI of 4 at which the leaf area per plant was nearly equivalent to that of DH618. The determination coefficient (*R*^2^), root mean square error (RMSE), and normalized RMSE (nRMSE) were applied to evaluate the agreement between the simulations and observations and can be calculated as follows:

$$R^{2}=\frac{\sum_{i=1}^{n}\;\left(O_{i}-\bar{O}\right)\left(S_{i}-\bar{S}\right)}{\sqrt{\sum_{i=1}^{n}\;{\left(O_{i}-\bar{O}\right)}^{2}\sum_{i=1}^{n}\;{\left(S_{i}-\bar{S}\right)}^{2}}}$$ (27)

$$\text{RMSE}=\sqrt{\frac{1}{n}\sum_{i=1}^{n}\;{\left(s_{i}-o_{i}\right)}^{2}}$$ (28)

$$\text{nRMSE}=\frac{\text{RMSE}}{\bar{O}}$$ (29)

where *O_i_* represents the observed value, *S_i_* represents the simulated value, $\bar{O}$ represents the mean value of the observations, $\bar{S}$ represents the mean value of the simulations, and *n* represents the sample size. The R language was used for model programming, scenario simulation, nonlinear regression, and statistical analysis.

## Results

### Simulated dry matter accumulation and RUE of maize canopies in relation to the LIA

The simulated ADM and RUE over the postsilking period first increased, then reached a maximum, and finally decreased with increasing leaf inclination across the scenarios with different LAIs (Table 2). The ADM reached its maximum at 1,978.51 ± 17.35, 2,056.40 ± 27.16, 2,046.46 ± 36.09, 1,987.93 ± 43.79, and 1,903.84 ± 51.00 g·m^−2^ when the leaf inclination distribution was A22 (63.48°), A23 (66.06°), A24 (68.62°), A25 (71.08°), and A25 (71.08°), respectively, for LAIs of 3, 4, 5, 6, and 7. The RUE over the postsilking period showed a similar pattern as a function of the leaf inclination as did the ADM. The RUE reached its maximum at 3.82 ± 0.03, 3.71 ± 0.05, 3.57 ± 0.06, 3.4 ± 0.08, and 3.22 ± 0.09 g·MJ^−1^ for A25 consistently across maize canopies with LAIs ranging from 3 to 7.

**Table 2.** Simulated dry matter accumulation and RUE of maize canopies in relation to the LIA

LIA	Mean value	ADM (g·m^−2^)					RUE (g·MJ^−1^)				
		3	4	5	6	7	3	4	5	6	7
1	13.30°	1,727.87 ± 15.83	1,748.02 ± 25.17	1,703.54 ± 33.56	1,630.79 ± 40.61	1,546.83 ± 46.76	3.12 ± 0.03	3.01 ± 0.04	2.87 ± 0.06	2.72 ± 0.07	2.57 ± 0.08
2	14.96°	1,741.80 ± 16.00	1,761.97 ± 25.43	1,716.85 ± 33.89	1,643.16 ± 41.00	1,558.16 ± 47.20	3.15 ± 0.03	3.03 ± 0.04	2.89 ± 0.06	2.74 ± 0.07	2.58 ± 0.08
3	16.94°	1,758.63 ± 16.21	1,778.95 ± 25.73	1,733.12 ± 34.28	1,658.30 ± 41.47	1,572.05 ± 47.72	3.18 ± 0.03	3.06 ± 0.04	2.92 ± 0.06	2.76 ± 0.07	2.61 ± 0.08
4	18.92°	1,774.68 ± 16.40	1,795.29 ± 26.01	1,748.87 ± 34.65	1,673.02 ± 41.91	1,585.57 ± 48.22	3.21 ± 0.03	3.09 ± 0.05	2.95 ± 0.06	2.79 ± 0.07	2.63 ± 0.08
5	21.38°	1,792.96 ± 16.61	1,814.16 ± 26.33	1,767.22 ± 35.06	1,690.27 ± 42.40	1,601.46 ± 48.77	3.25 ± 0.03	3.13 ± 0.05	2.98 ± 0.06	2.82 ± 0.07	2.66 ± 0.08
6	23.94°	1,810.33 ± 16.81	1,832.46 ± 26.61	1,785.23 ± 35.42	1,707.32 ± 42.84	1,617.26 ± 49.27	3.29 ± 0.03	3.16 ± 0.05	3.01 ± 0.06	2.85 ± 0.07	2.68 ± 0.08
7	26.52°	1,826.65 ± 16.97	1,850.03 ± 26.85	1,802.03 ± 35.74	1,724.10 ± 42.23	1,632.88 ± 49.73	3.32 ± 0.03	3.19 ± 0.05	3.04 ± 0.06	2.88 ± 0.07	2.71 ± 0.08
8	29.34°	1,843.60 ± 17.13	1,868.73 ± 27.08	1,821.75 ± 36.05	1,742.42 ±43.62	1,650.06 ± 50.18	3.36 ± 0.03	3.23 ± 0.05	3.07 ± 0.06	2.91 ± 0.07	2.74 ± 0.08
9	31.65°	1,856.87 ± 17.23	1,883.73 ± 27.23	1,837.21 ± 36.26	1,757.52 ± 43.89	1,664.31 ± 50.51	3.39 ± 0.03	3.26 ± 0.05	3.1 ± 0.06	2.93 ± 0.07	2.76 ± 0.08
10	33.84°	1,869.07 ± 17.32	1,897.86 ± 27.36	1,851.99 ± 36.44	1,772.08 ± 44.13	1,678.16 ± 50.81	3.42 ± 0.03	3.29 ± 0.05	3.13 ± 0.06	2.96 ± 0.07	2.79 ± 0.08
11	36.49°	1,883.29 ± 17.41	1,914.75 ± 27.49	1,869.95 ± 36.62	1,789.98 ± 44.38	1,695.30 ± 51.12	3.45 ± 0.03	3.32 ± 0.05	3.16 ± 0.06	2.99 ± 0.07	2.82 ± 0.09
12	39.06°	1,896.70 ± 17.48	1,931.12 ± 27.59	1,887.67 ± 36.77	1,807.85 ± 44.59	1,712.56 ± 51.40	3.49 ± 0.03	3.36 ± 0.05	3.2 ± 0.06	3.02 ± 0.08	2.85 ± 0.09
13	41.65°	1,909.77 ± 17.53	1,947.56 ± 27.66	1,905.79 ± 36.89	1,826.37 ± 44.76	1,730.60 ± 51.64	3.53 ± 0.03	3.39 ± 0.05	3.23 ± 0.06	3.06 ± 0.08	2.88 ± 0.09
14	43.74°	1,919.92 ± 17.56	1,960.71 ± 27.70	1,920.54 ± 36.96	1,841.62 ± 44.88	1,745.60 ± 51.81	3.55 ± 0.03	3.42 ± 0.05	3.26 ± 0.06	3.08 ± 0.08	2.9 ± 0.09
15	46.27°	1,931.74 ± 17.58	1,976.47 ± 27.73	1,938.55 ± 37.01	1,860.48 ± 44.99	1,764.32 ± 51.98	3.59 ± 0.03	3.46 ± 0.05	3.3 ± 0.06	3.12 ± 0.08	2.94 ± 0.09
16	48.35°	1,940.93 ± 17.59	1,989.13 ± 27.74	1,953.32 ± 37.04	1,876.17 ± 45.05	1,780.05 ± 52.10	3.62 ± 0.03	3.49 ± 0.05	3.33 ± 0.06	3.15 ± 0.08	2.97 ± 0.09
17	50.94°	1,951.40 ± 17.58	2,004.16 ± 27.73	1,971.25 ± 37.04	1,895.53 ± 45.09	1,799.69 ± 52.20	3.66 ± 0.03	3.53 ± 0.05	3.37 ± 0.06	3.19 ± 0.08	3 ± 0.09
18	53.51°	1,960.57 ± 17.57	2,018.03 ± 27.69	1,988.31 ± 37.00	1,914.31 ± 45.09	1,819.03 ± 52.25	3.69 ± 0.03	3.56 ± 0.05	3.4 ± 0.06	3.22 ± 0.08	3.04 ± 0.09
19	56.16°	1,968.39 ± 17.53	2,030.79 ± 27.63	2,004.62 ± 36.93	1,932.71 ± 45.04	1,838.31 ± 52.24	3.72 ± 0.03	3.6 ± 0.05	3.44 ± 0.06	3.26 ± 0.08	3.08 ± 0.09
20	58.35°	1,973.39 ± 17.49	2,039.87 ± 27.55	2,016.78 ± 36.85	1,946.85 ± 44.96	1,853.43 ± 52.19	3.75 ± 0.03	3.63 ± 0.05	3.47 ± 0.06	3.29 ± 0.08	3.11 ± 0.09
21	60.66°	1,976.92 ± 17.43	2,047.55 ± 27.46	2,027.78 ± 36.73	1,960.10 ± 44.84	1,867.96 ± 52.09	3.77 ± 0.03	3.65 ± 0.05	3.5 ± 0.06	3.32 ± 0.08	3.14 ± 0.09
22	63.48°	1,978.51 ± 17.35	2,053.88 ± 27.32	2,038.13 ± 36.54	1,973.41 ± 44.63	1,883.13 ± 51.90	3.8 ± 0.03	3.68 ± 0.05	3.53 ± 0.06	3.35 ± 0.08	3.17 ± 0.09
23	66.06°	1,977.15 ± 17.26	2,056.40 ± 27.16	2,044.19 ± 36.33	1,982.26 ± 44.40	1,893.93 ± 51.65	3.81 ± 0.03	3.7 ± 0.05	3.55 ± 0.06	3.38 ± 0.08	3.19 ± 0.09
24	68.62°	1,972.91 ± 17.15	2,055.42 ± 26.98	2,046.46 ± 36.09	1,987.30 ± 44.11	1,901.08 ± 51.35	3.82 ± 0.03	3.71 ± 0.05	3.56 ± 0.06	3.39 ± 0.08	3.21 ± 0.09
25	71.08°	1,965.75 ± 17.03	2,050.72 ± 26.78	2,044.54 ± 35.82	1,987.93 ± 43.79	1,903.84 ± 51.00	3.82 ± 0.03	3.71 ± 0.05	3.57 ± 0.06	3.4 ± 0.08	3.22 ± 0.09
26	73.06°	1,957.55 ± 16.92	2,043.95 ± 26.60	2,039.68 ± 35.58	1,985.02 ± 43.50	1,902.66 ± 50.67	3.81 ± 0.03	3.71 ± 0.05	3.57 ± 0.06	3.4 ± 0.07	3.22 ± 0.09
27	75.04°	1,947.26 ± 16.79	2,043.57 ± 26.41	2,031.86 ± 35.31	1,978.97 ± 43.17	1,898.33 ± 50.30	3.80 ± 0.03	3.70 ± 0.05	3.56 ± 0.06	3.39 ± 0.07	3.22 ± 0.09
28	76.70°	1,937.09 ± 16.68	2,024.56 ± 26.24	2,022.71 ± 35.08	1,971.01 ± 42.89	1,891.65 ± 49.98	3.79 ± 0.03	3.68 ± 0.05	3.54 ± 0.06	3.38 ± 0.07	3.21 ± 0.08

### Simulated dry matter accumulation and RUE of a maize canopy in response to the leaf nitrogen and leaf area vertical distributions

**Fig. 4.**
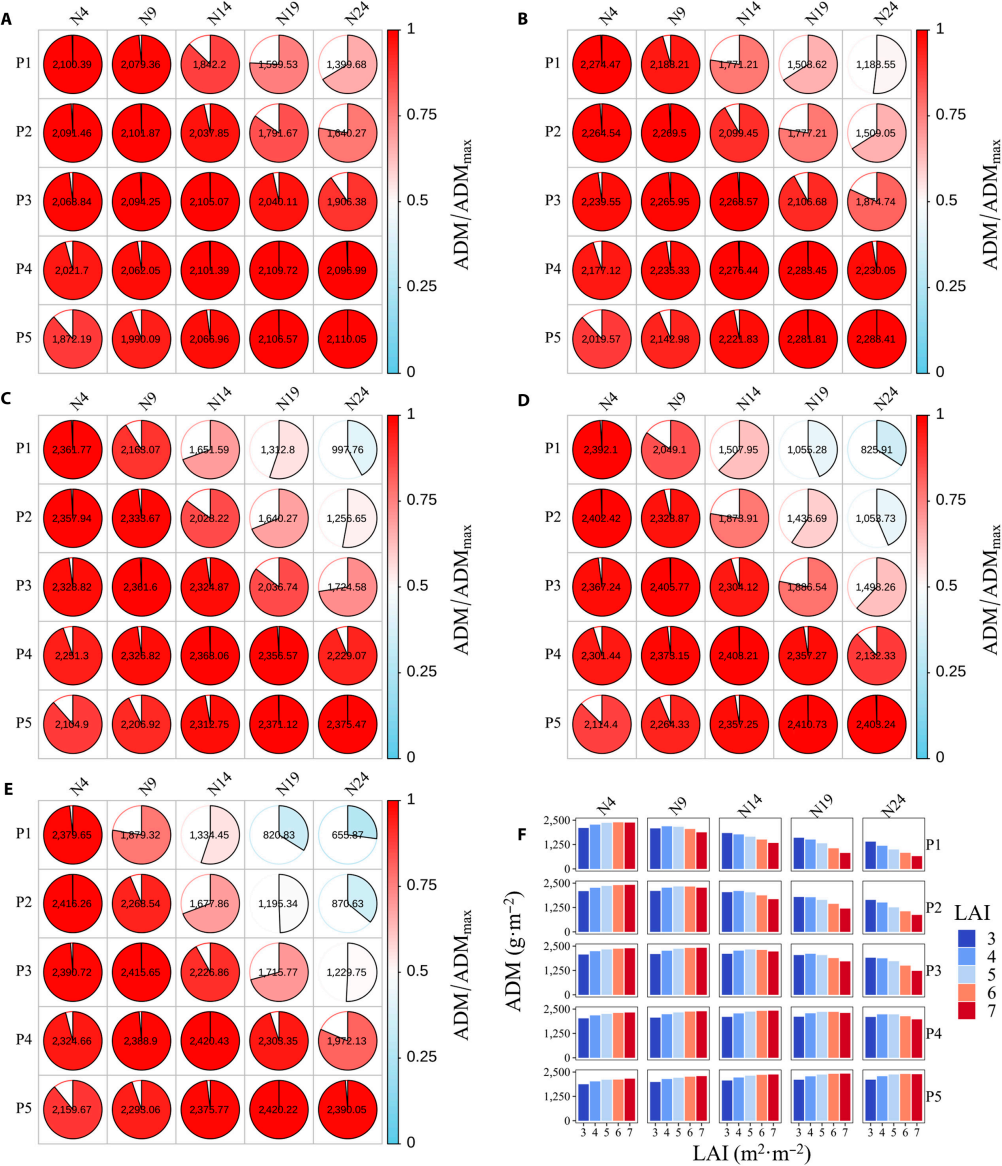
Simulated effects of the leaf area and leaf nitrogen vertical distributions on the ADM of the maize canopy at LAIs of 3 (A), 4 (B), 5 (C), 6 (D), and 7 (E). The normalized ADM, which was normalized by its maximal value for maize canopies with different LAIs, is denoted by partially filled pie charts with color gradients. Both the filled portion and color gradient indicate the magnitude of the normalized ADM. The ADM value is also labeled in the middle of each partially filled pie chart. The colored columns denote the simulated ADM for maize canopies with different LAIs (F). P1 and N4, P2 and N9, P3 and N14, P4 and N19, and P5 and N24 shared similar vertical distribution patterns in which the maximum total leaf area and leaf nitrogen fractions were achieved in the upper, middle-upper, middle, middle-lower, and lower canopies, respectively.

**Fig. 5.**
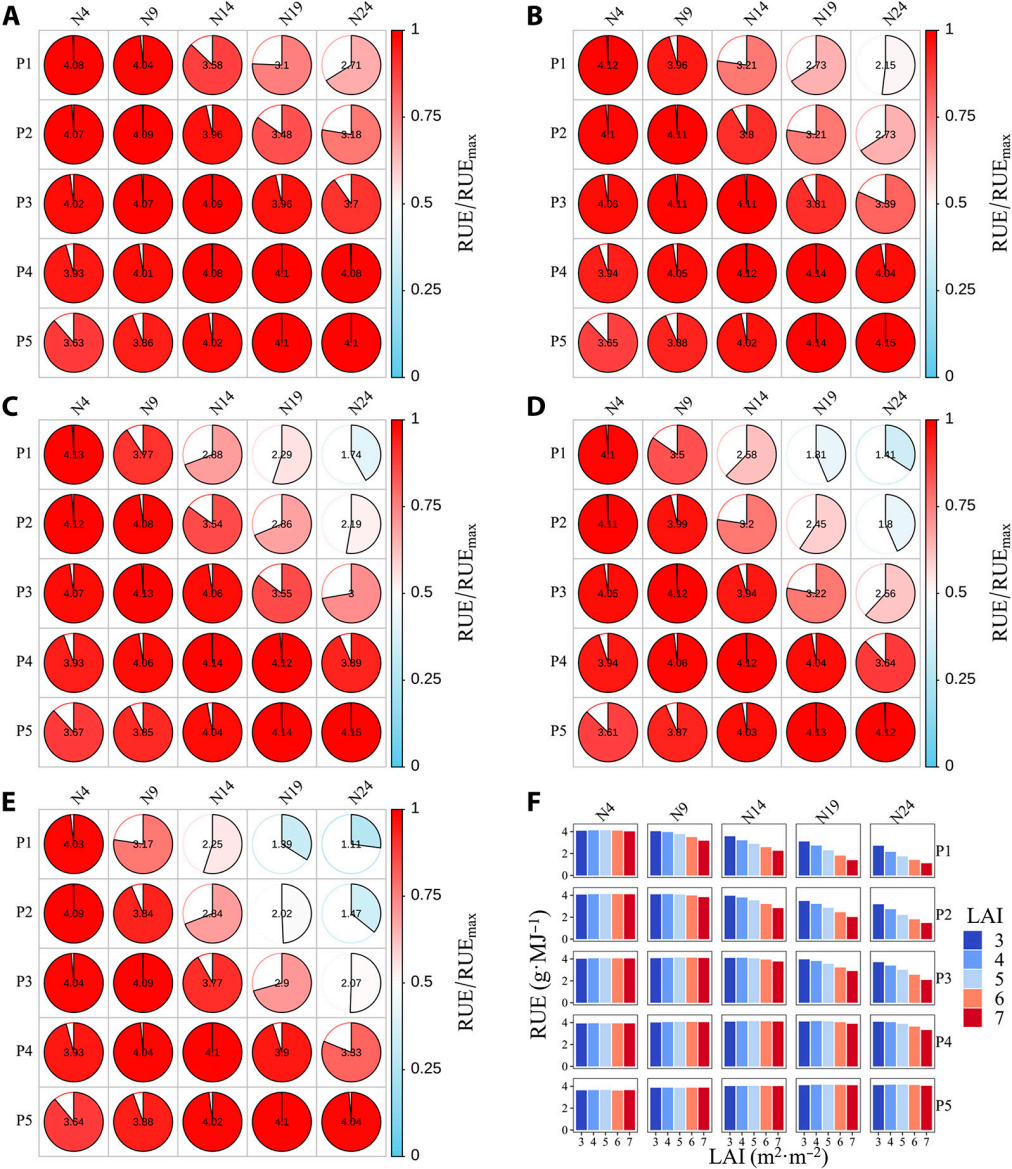
Simulated effects of the leaf area and leaf nitrogen vertical distributions on the RUE of the maize canopy at LAIs of 3 (A), 4 (B), 5 (C), 6 (D), and 7 (E). The normalized RUE, which was normalized by its maximal value for maize canopies with different LAIs, is denoted by partially filled pie charts with color gradients. Both the filled portion and color gradient indicate the magnitude of the normalized RUE. The RUE values are also labeled in the middle of each partially filled pie chart. The colored columns denote the simulated RUE for maize canopies with different LAIs (F). P1 and N4, P2 and N9, P3 and N14, P4 and N19, and P5 and N24 shared similar vertical distribution patterns in which the maximum total leaf area and leaf nitrogen fractions were both achieved in the upper, middle-upper, middle, middle-lower, and lower canopies, respectively.

The simulated ADM and RUE of the maize canopy after silking under different combinations of the leaf nitrogen and leaf area distributions showed large variations, with relative values ranging from 0.63, 0.48, 0.38, 0.30, and 0.21 to 1 in scenarios with LAIs of 3, 4, 5, 6, and 7, respectively (Figs. S1 and S2). For canopies with a vertical leaf area pattern of P1, P2, P3, P4, and P5, the simulated ADM and RUE reached their maximum values at N1, N9, N13, N18, and N23, respectively, when the LAI was 3 (Figs. S1A and S2A); at N1, N8, N12, N17, and N23, when the LAI was 4 (Figs. S1B and S2B); at N1, N6, N10, N16, and N22, when the LAI was 5 (Figs. S1C and S2C); at N1, N5, N9, N15, and N21, when the LAI was 6 (Figs. S1D and S2D); and at N1, N4, N8, N14, and N20, when the LAI was 7 (Figs. S1E and S2E). The ADM and RUE reached their maximum values when the vertical distribution of the leaf area matched that of the leaf nitrogen content (Figs. 4A to E and 5A to E). The average ADM concentrations were 2,231.33, 2,219.53, 2,078.53, 1,876.99, and 1,674.38 g·m^−2^, and the average RUEs were 3.96, 3.94, 3.70, 3.35, and 2.99 g·MJ^−1^ for N4, N9, N14, N19, and N24, respectively, across canopies with different LAIs (Figs. 4E and Fig. 5E), indicating that allocating more leaf nitrogen to the upper canopy results in greater dry matter accumulation and RUE irrespective of the leaf area distribution. The average ADM concentrations were 1,621.99, 1,831.62, 2,050.93, 2,226.31, and 2,221.92 g·m^−2^, and the average RUEs were 2.90, 3.27, 3.65, 3.96, and 3.95 g·MJ^−1^ for P1, P2, P3, P4, and P5, respectively, across canopies with different leaf nitrogen distributions at different LAIs (Figs. S1 and S2), indicating that canopies with more leaf area in the lower part produce more dry matter and thus result in a higher RUE irrespective of the leaf nitrogen distribution.

### Validation and optimization of the ideotype canopy in maize

**Fig. 6.**
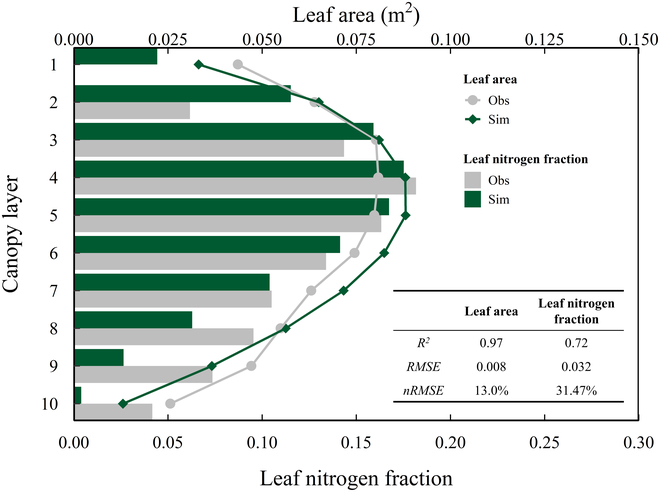
The vertical distributions of the leaf area and leaf nitrogen fractions for the actual high-yield cultivar DH618 in Xinjiang in 2015 and for an optimal canopy from simulations. The solid lines with different colors represent the observations (gray) and simulations (green) of the vertical leaf area distribution. Filled bars with different colors represent the observations (gray) and simulations (green) of the vertical leaf nitrogen distribution.

**Fig. 7.**
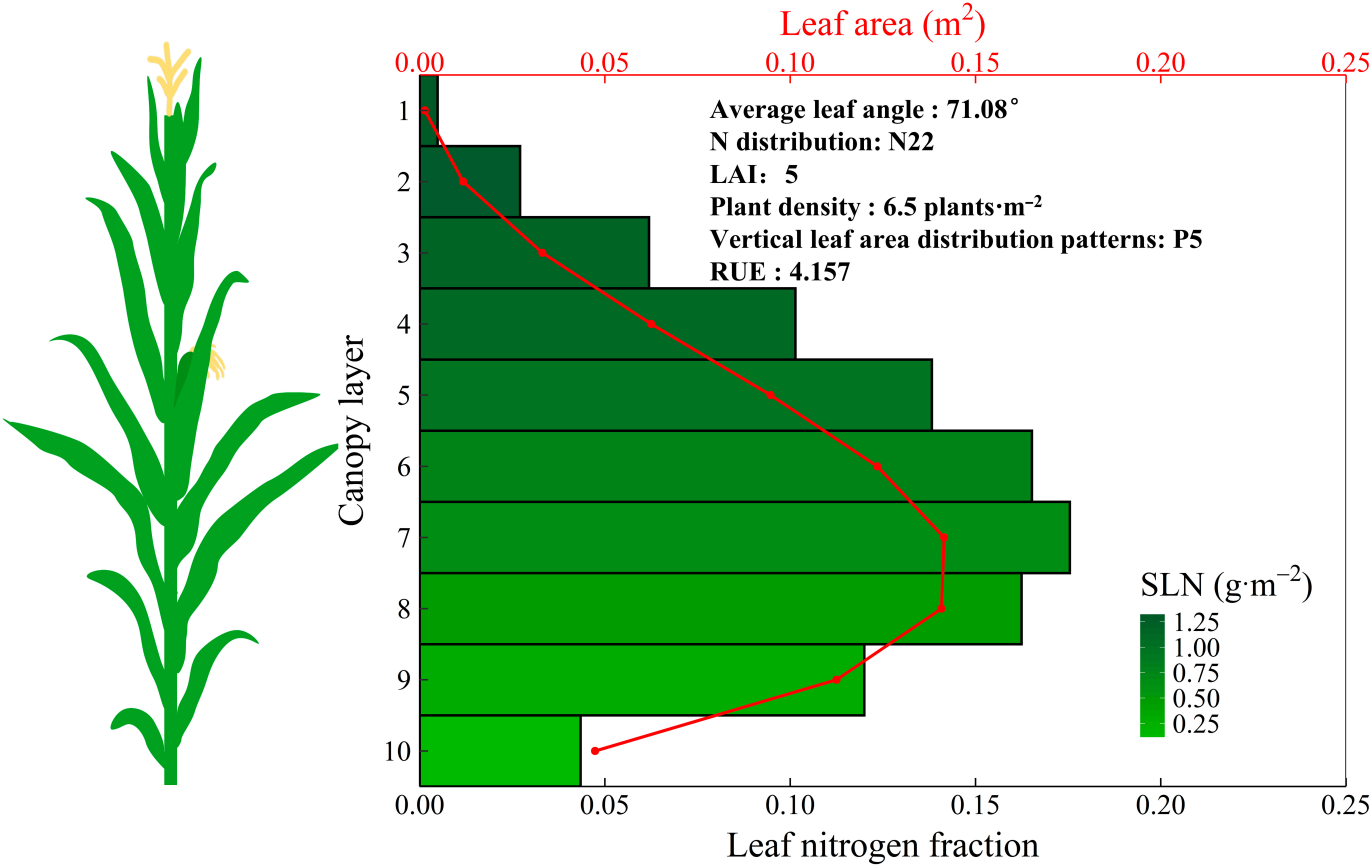
Maize ideotype for maximizing the RUE. The red solid line represents the vertical leaf area distribution, bars represent the leaf nitrogen fraction, and color gradients represent the SLN at different canopy layers.

The simulated optimal vertical distributions of the leaf area and leaf nitrogen fraction in the maize canopy agreed well with the field experiment observations. The overall *R*^2^ values were 0.97 and 0.72 for the leaf area and leaf nitrogen fraction, respectively, and the nRMSE values were 13.00% and 31.47%, respectively (Fig. 6). The leaf area and leaf nitrogen fractions followed a similar beta-shaped pattern with increasing depth from the top to the bottom of the canopy, suggesting that the RUE can be maximized by matching the vertical distributions of the leaf area and leaf nitrogen in the canopy. Across all scenarios, the maximum RUE (4.157 g·MJ^−1^) was achieved by the ideotype canopy with an LAI of 5, an average leaf angle of 71.08°, a beta-shape vertical leaf area, and a nitrogen distribution with a peak at the seventh canopy layer (Fig. 7). The SLN gradually decreased from the canopy top to the bottom (Fig. 7).

### The optimal vertical distributions of light and nitrogen in relation to the LAI and the vertical leaf area distribution

**Fig. 8.**
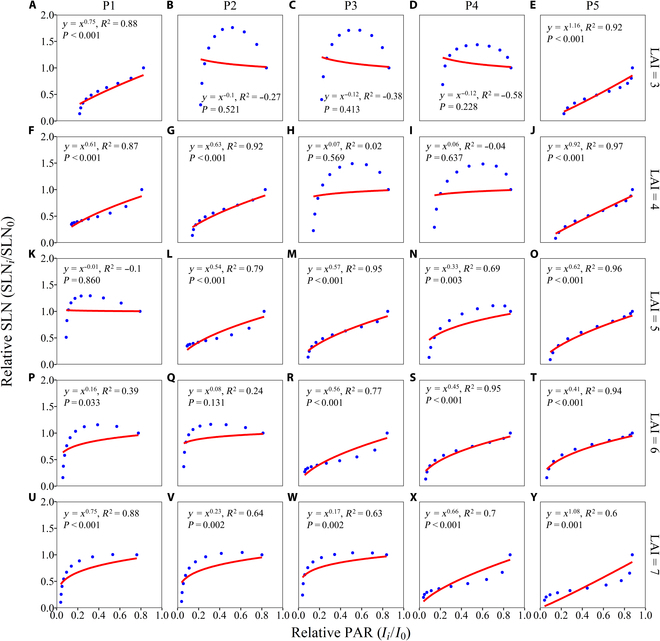
Relationships between the relative SLN and relative PAR for the optimal vertical leaf nitrogen distribution under which the maximum RUE is achieved. Light-SLN relationships for simulated maize canopies with different vertical leaf area distribution patterns (P1 to P5) at LAIs of 3 (A to E), 4 (F to J), 5 (K to O), 6 (P to T), and 7 (U to Y) are shown.

To examine whether the canopy RUE can be maximized by the coordination of light and nitrogen vertical distributions in maize canopies with different LAIs and leaf area distributions, we plotted the SLN (expressed using relative values normalized by the SLN of the top layer in the canopy) against PAR (expressed using relative values normalized by the PAR of the top layer in the canopy) for the scenario in which the maximum simulated RUE was achieved (Fig. 8). By utilizing nonlinear fitting methods to obtain parameter estimates, this study aimed to evaluate the light-SLN matching ability of canopies. The power equation (Eq. 26) fit well for most canopies with different LAIs and leaf area distributions except for canopies with lower LAIs and relatively more leaf area located at lower positions in the canopy and with higher LAIs and relatively more leaf area located at higher positions, where the relative SLN showed a parabolic relationship with the relative PAR (Figs. 8B to D, H, I, P, and Q). The *K*_N_*/K*_L_ ratios on average were 0.955, 0.720, 0.515, 0.658, and 0.478 with increasing LAI from 3 to 7 by excluding the nonsignificant fits (Fig. 8). The most proximity of *K*_N_*/K*_L_ to 1 was achieved when relatively more leaf area was located at a lower position in the canopy across a wide range of LAIs (Fig. 8E, J, O, and Y).

## Discussion

### The LIA of maize ideotypes that maximizes the RUE

Significant genetic progress has been made in improving morphological and photosynthetic traits to increase maize yield and the RUE [18,36,53,54]. Over the past few decades, there has been a notable increase in the leaf angle of released maize hybrids, leading to optimized light distribution within the canopy and consequently increased RUEs [36,54]. Li et al. [55] categorized maize yield from a multiyear cultivar field experiment into 4 levels and observed that the RUE increased, while the leaf angle above the ear decreased with increasing yield. In this study, the LIA of the maize ideotype increased from 63.48° to 71.08° as the LAI increased from 3 to 7 (Table 2). This finding is consistent with Duncan’s simulations, which demonstrated that a larger LIA had a more significant effect on the light use efficiency of maize at leaf area indices above 4 [56]. Duncan [57] suggested that an optimum LIA of 70° could achieve high yields in maize. Similarly, Liu et al. [52] observed high yields in maize with LIAs greater than 73.3° above the ear and 64.9° below the ear, with an average LIA of 69.1°, which is consistent with our findings. The RUE in maize was reported to reach a maximum at LIAs of 74° [55], 73.6° [58], and 72° [54], confirming our simulations at 71.08°. Overall, our simulations provided a valid LIA for achieving a greater photosynthetic efficiency and productivity in maize.

### Maximizing the RUE by matching the leaf area and leaf nitrogen of canopy layers

Breeding efforts in maize have focused not only on increasing the total leaf area per plant but also on modifying the vertical distribution of the leaf area within the canopy [11,17,55,59]. A larger fraction of the LAI was found to be concentrated in the upper canopy for modern cultivars with higher yields and RUEs [18,52,55]. The simulated LAI distribution with an emergent maximum RUE was in good agreement with the experimental observations, in which more leaf area was located in the upper canopy and the leaf area per individual layer reached a maximum in the fourth layer (i.e., a relative canopy height of 0.6) (Fig. 6). Canopy photosynthesis in maize can be enhanced by optimizing the leaf area distribution with a suppressed decreasing gradient in a constant LAI scenario according to a simulation study by Stewart et al. [60], which corresponds qualitatively with our simulations. Because more leaves of a compact plant in an open canopy are likely under sunlit light conditions, a modification of the leaf area distribution, as mentioned above, could allow more light to penetrate into larger leaves in the lower canopy, thereby increasing canopy photosynthesis.

The vertical distribution of leaf nitrogen is determined by genetic factors and local light availability [48,61]. Leaf SLN was found to decrease toward the canopy bottom, which paralleled the trend of light [12,62]. Our results showed that both yield and the RUE can be maximized when the vertical distribution of the leaf nitrogen content within the canopy matches that of the LAI (Figs. 4 and 5). Liu et al. [59] reported a significant increase in the RUE of modern cultivars compared to older cultivars, possibly because of rapid leaf growth, a compact plant type, and a greater leaf area in the middle canopy across different nitrogen supplies. A similar increasing pattern of the RUE against the year of commercialization in US maize cultivars was also documented by Reynolds and Langridge [28], who partially attributed this to the delayed leaf senescence resulting from the positive trend in the SLN for canopy strata above and below the ear. The adjustments of the leaf nitrogen partitioning pattern to maximize the RUE resulting from our simulations are consistent with the conceptual framework for improving the maize RUE by Lacasa et al. [11].

### Explanations for the discrepancy between our simulated and optimal light-SLN profiles

Our results indicated that to maximize the RUE in maize, a greater proportion of leaf nitrogen is preferentially allocated to canopy strata with higher LAIs (Figs. 4 and 5). However, our simulations also demonstrated that the light gradient pattern is not necessarily parallel to the distribution of the leaf SLN, and the degree of matching depends heavily on the leaf area profile (Fig. 8). The mismatch between light and the SLN occurs primarily in canopies with lower total leaf areas, but there is a clear coordination between the leaf area and SLN, suggesting the prominent contribution of the leaf area to canopy photosynthetic production in canopies with a uniform light distribution. This finding is partly consistent with the experimental study by Liu et al. [59], who reported a substantial increase in the leaf area across canopy layers, along with a significant decrease in both the leaf angle and leaf orientation, resulting in an increase in the RUE regardless of the nitrogen proportion. An increase in the leaf area leads to a larger photosynthetic area but a lower photosynthetic capacity (i.e., SLN) when the leaf nitrogen is fixed, indicating the need to optimize the leaf area and its distribution to balance this trade-off. Modifying the leaf area distribution is beneficial for optimizing light interception and thereby increasing the apparent photosynthetic use efficiency of light, particularly under high light intensities [63]. Our results also agree well with those of Goudriaan [64], who concluded that optimization theory is most applicable to canopies with higher values. This is because the simulations for scenarios combining the vertical distribution of leaf nitrogen partitioning and LAI were performed under the optimal LIA, allowing for noticeable light saturation in the middle canopy, especially in open canopies. Greater SLN values in small and light-saturated leaves in the upper canopy had relatively little effect on canopy photosynthesis. The greater leaf nitrogen allocation in the middle canopy stratum with greater leaf area and less allocation to the light-saturated canopy top and shaded canopy bottom with lower LAI are consistent with the observed patterns in high-photosynthesis maize cultivars in terms of partitioning and reallocation [36].

### Potential limitations and future directions

This study used computational analyses to generate various scenarios by combining the leaf area and leaf nitrogen vertical distributions, and it is acknowledged that some of these distributions may not accurately represent actual crops. However, such computational analyses are valuable for testing hypotheses and providing benchmarks. For example, the presence of a greater leaf nitrogen fraction in the lower canopy leaves, as generated in this study, may not always be the case during the late grain-filling stage in maize due to the upward leaf nitrogen remobilization to grains [36]. However, this distribution indicates greater potential for the photosynthetic use of intercepted light and can help mitigate the risk of photoinhibition under bright sunflecks, especially in an open canopy where lower leaves are exposed to light [65]. The simplification of not considering temporal changes in the leaf nitrogen content over canopy layers in the model simulations may lead to an overestimation of the RUE during the postsilking period, as there is a decrease in photosynthetic capacity across canopy layers due to the translocation of nitrogen from leaves to grains. However, studies have shown that the pattern of the vertical distribution of leaf nitrogen partitioning is not significantly altered [48], suggesting that the ideotype proposed in the present study may still be valid. The leaf nitrogen concentration and content have been shown to change both vertically and horizontally over time [19], whereas this was scarcely considered in previous studies and this study when characterizing the light-SLN profile for maximizing the RUE. Moreover, the leaf nitrogen allocation in different forms has been shown to have a substantial impact on canopy photosynthesis [66–68] and has been suggested as an effective strategy for improving photosynthetic production [69]. The spatiotemporal distribution of leaf nitrogen in both the vertical and horizontal directions across the canopy should be considered in future studies, and the rate of nitrogen translocation from leaves could be another target trait for optimizing the canopy RUE. The advancement of phenotyping technology has made it possible to determine structural and functional traits at the facet level [70] and to obtain leaf nitrogen profiles using unmanned aerial vehicles at the canopy level [71]. Its further integration into the canopy photosynthesis model could not only facilitate explicit and precise profiling of the ideotype for maximizing the RUE but also be a primary step toward high-throughput phenotyping and screening of the RUE for massive numbers of inbred lines and cultivars.

## Conclusion

The concept of maximizing canopy photosynthetic production through matching the vertical distribution of light and the leaf nitrogen content has long been recognized. To translate this theory into practical applications from an agronomic and breeding perspective, we developed numerous virtual canopies to cover a wide range of canopy structural and functional traits by considering the LIA, LAI, leaf area distribution, and leaf nitrogen allocation pattern. The ADM and RUE were simulated by an improved multilayer canopy photosynthesis model. The strategy of matching the leaf area and leaf nitrogen vertically in the canopy proved to be effective in improving the RUE in maize across different scenarios. In addition, the pattern of light-SLN coordination based on optimization theory emerged as a property from the simulations to maximize the RUE in most scenarios, particularly in dense canopies. These results suggest that matching the leaf area to leaf nitrogen content vertically in the canopy may be a robust and practical strategy for maximizing canopy photosynthetic production and the RUE in maize.

## Data Availability

The data used to support the findings of this study are available from the corresponding author upon reasonable request.
