# Supplementary figures and images for: Maximizing the Radiation Use Efficiency by Matching the Leaf Area and Leaf Nitrogen Vertical Distributions in a Maize Canopy: A Simulation Study
Source: Plant Phenomics. 2024 Jul 29;6:0217. doi: 10.34133/plantphenomics.0217 (PMC11284131; doi:10.34133/plantphenomics.0217)

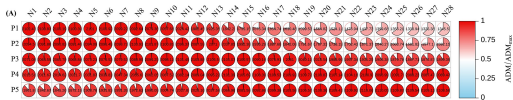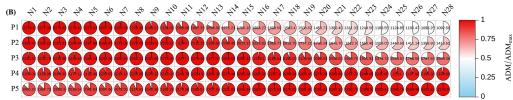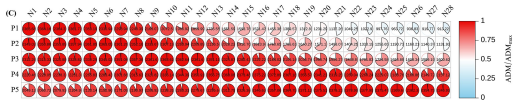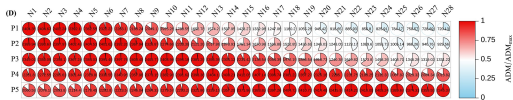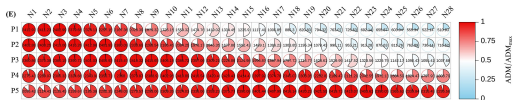

Supplement: Supplementary 1 — Tables S1 to S3 Figs. S1 and S2 [file plantphenomics.0217.f1.zip › Fig.S1.pdf]

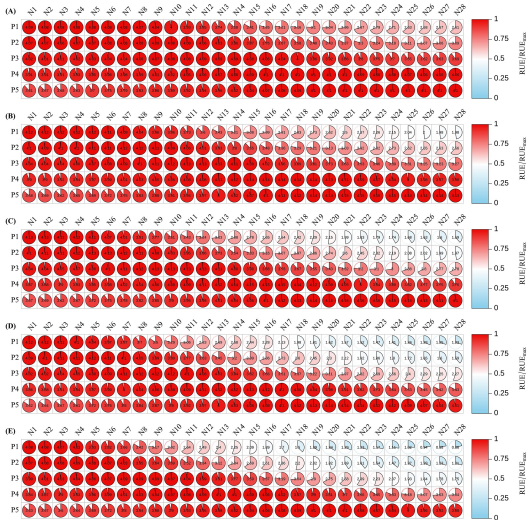

Supplement: Supplementary 1 — Tables S1 to S3 Figs. S1 and S2 [file plantphenomics.0217.f1.zip › Fig.S2.pdf]
