# Supplementary material for: Maximizing the Radiation Use Efficiency by Matching the Leaf Area and Leaf Nitrogen Vertical Distributions in a Maize Canopy: A Simulation Study
Source: Plant Phenomics. 2024 Jul 29;6:0217. doi: 10.34133/plantphenomics.0217 (PMC11284131; doi:10.34133/plantphenomics.0217)
Supplement: Supplementary 1 — Tables S1 to S3 Figs. S1 and S2 [file plantphenomics.0217.f1.zip › RevisedSupplementaryFile_PlantPhenomics-D-24-00002.doc]

Table S1 Description of symbols that were used in the canopy photosynthesis model

| **Symbol** | **Description** | **Units** | **Modules** | **Value** | **Equation** |
| --- | --- | --- | --- | --- | --- |
| *K_dir(t)_* | Extinction coefficient of direct PAR at the time *t* | dimensionless | Light distribution | - | (1), (7), (14) |
| *O_av_* | Average projection of the leaves in the direction of the solar beam | dimensionless | Light distribution | - | (1), (3) |
| *O_av(a)_* | Solar projection at the *a*th leaf inclination angle class | dimensionless | Light distribution | - | (2), (3) |
| *β_(t)_* | Solar altitude angle at time *t* of the day | ° | Light distribution | - | (1), (2), (18) |
| *β_L(a)_* | Leaf inclination angle | ° | Light distribution | - | (2) |
| *f_a_* | Leaves angle frequency | dimensionless | Light distribution | - | (3), (4) |
| *f(b)* | Probability density of leaf inclination | dimensionless | Light distribution | - | (4) |
| *μ, ν* | Parameters of beta-distribution | dimensionless | Light distribution | - | (5), (6) |
| *I*_dir_*_(0,t)_* | Direct incident PAR at the hour *t* of day | μmol photons m^-2^ s^-1^ | Light distribution | - | (7) |
| *I*_diff_*_(0,t)_* | Diffuse incident PAR at the hour *t* of day | μmol photons m^-2^ s^-1^ | Light distribution | - | (8) |
| *I*_dir_*_(n,t)_* | Direct PAR per unit ground area at the top of the *n*th canopy layer at the hour *t* of the day | μmol photons m^-2^ s^-1^ | Light distribution | - | (7), (10) |
| *I*_diff_*_(n,t)_* | Diffuse PAR per unit ground area at the top of the *n*th canopy layer at the hour *t* of the day | μmol photons m^-2^ s^-1^ | Light distribution | - | (8), (11) |
| *K*_diff_ | PAR extinction coefficient for diffuse | dimensionless | Light distribution | 0.7[1] | (8) |
| *CLAI_n_* | Cumulative leaf area index at the top of the *n*th canopy layer | m^2^ m^-2^ | Light distribution | - | (7), (8), (9), (14) |
| *LAI* | Leaf area index of the canopy | m^2^ m^-2^ | Light distribution | 3, 4, 5, 6, 7 | (9) |
| *Z_e_* | Total canopy depth of maize | m | Light distribution | - | (9) |
| *Z_m_* | Canopy depth at a given canopy interval where the corresponding LAI reaches the maximum | m | Light distribution | - | (9) |
| *Z_n_* | Canopy depth at the bottom of the *n*th canopy layer that is measured from the top downwardly | m | Light distribution | - | (9) |
| *I*_PAR_*_(t)_* | Intercepted PAR by the entire canopy at the *t* time of the day | μmol photons m^-2^ s^-1^ | Light distribution | - | (12), (13) |
| *I*_PAR_*_(DAY)_* | Intercepted PAR by the entire canopy over the day | MJ m^-2^ d^-1^ | Light distribution | - | (13) |
| *f*_sun_*_(n,t)_* | Fraction of sunlit leaves in the nth canopy at the hour *t* of the day | dimensionless | Canopy photosynthesis | - | (14), (15) |
| *f*_sh_*_(n,t)_* | Fraction of shaded in the *n*th canopy at the hour t of the day | dimensionless | Canopy photosynthesis | - | (15) |
| *A*_sun_*_(n,a,t)_* | Instantaneous photosynthetic rates of sunlit leaves at the time *t* of the *a*th leaves inclination class of the *n*th canopy layer | μmol CO_2_ m^-2^ leaf s^-1^ | Canopy photosynthesis | - | (16) |
| *A*_sh_*_(n,t)_* | Instantaneous photosynthetic rates of shade leaves at time t of the nth canopy of the day | μmol CO_2_ m^-2^ leaf s^-1^ | Canopy photosynthesis | - | (17) |
| *I*_sun_*_(n,a,t)_* | Intercepted PAR per unit leaf area of the sunlit leaves of *a*th leaves inclination class of the *n*th canopy of the day at time *t* | μmol CO_2_ m^-2^ leaf s^-1^ | Canopy photosynthesis | - | (16), (18) |
| *I*_sh_*_(n,t)_* | Intercepted PAR per unit leaf area of the shaded leaves of the nth canopy of the day at time t | μmol CO_2_ m^-2^ leaf s^-1^ | Canopy photosynthesis | - | (18), (19) |
| *α* | Apparent quantum efficiency | μmol CO_2_ μmol-1 photons | Canopy photosynthesis | 0.05 | (16), (17) |
| *θ* | Empirical coefficient | dimensionless | Canopy photosynthesis | 0.8 | (16), (17) |
| *ζ_(a,t)_* | Leaf inclination angle relative to the direct radiation | ° | Canopy photosynthesis | - | (18) |
| *LAP_n_* | Leaf area per unit of land area of the *n*th individual canopy layer | m^2^ m^-2^ | Canopy photosynthesis | - | (19), (21), (23) |
| *A*_max_*_(n)_* | Maximum light-saturated leaf CO_2_ assimilation rate at *n*th canopy | μmol CO_2_ m^-2^ s^-1^ | Canopy photosynthesis | - | (20) |
| *SLN_n_* | Specific leaf nitrogen of the *n*th canopy layer | g m^-2^ | Canopy photosynthesis | - | (20), (22) |
| *LA_n_* | Leaf area at the *n*th canopy | m^2^ | Canopy photosynthesis | - | (21), (22) |
| *LNC_n_* | Leaf nitrogen content | mg | Canopy photosynthesis | 900 | (22) |
| *ρ* | Planting density | plants m^-2^ | Canopy photosynthesis | 6.5 | (21) |
| *A*_can_*_(DAY)_* | Daily canopy assimilation of CO_2_ | μmol CO_2_ m^-2^ d^-1^ | Canopy photosynthesis | - | (24) |
| *A*_can_*_(t)_* | Instantaneous photosynthetic rate for the entire canopy at time *t* of the day | μmol CO_2_ m^-2^ s^-1^ | Dry matter accumulation | - | (23), (24), (25) |
| *DM_(DAY)_* | Canopy daily accumulation of above-ground dry matter | g m^-2^ d^-1^ | Dry matter accumulation | - | (25) |
| *B* | Dry matter produced per unit mass of CO_2_ | g g-1 | Dry matter accumulation | 0.41[2] | (25) |

[1]	Bonelli, L.E. and Andrade, F.H. (2020) Maize Radiation Use-Efficiency Response to Optimally Distributed Foliar-Nitrogen-Content Depends on Canopy Leaf-Area Index. Field Crops Research, 247, 107557. https://doi.org/10.1016/j.fcr.2019.107557.

[2]	Sinclair, T.R. and Horie, T. (1989) Leaf Nitrogen, Photosynthesis, and Crop Radiation Use Efficiency: A Review. Crop Science, 29, 90-98. https://doi.org/10.2135/cropsci1989.0011183X002900010023x.

Table S2 The probability density of 28 leaf inclination distributions.

| Average leaf inclination angle | Leaf inclination angle frequency | | | | | | | | |
| --- | --- | --- | --- | --- | --- | --- | --- | --- | --- |
|  | 1-10° | 11-20° | 21-30° | 31-40° | 41-50° | 51-60° | 61-70° | 71-80° | 81-89° |
| 13.30° | 0.444 | 0.369 | 0.143 | 0.037 | 0.006 | 0.001 | 0.000 | 0.000 | 0.000 |
| 14.96° | 0.332 | 0.444 | 0.180 | 0.040 | 0.005 | 0.000 | 0.000 | 0.000 | 0.000 |
| 16.94° | 0.216 | 0.490 | 0.237 | 0.051 | 0.005 | 0.000 | 0.000 | 0.000 | 0.000 |
| 18.92° | 0.127 | 0.490 | 0.305 | 0.070 | 0.007 | 0.000 | 0.000 | 0.000 | 0.000 |
| 21.38 | 0.057 | 0.431 | 0.392 | 0.108 | 0.011 | 0.000 | 0.000 | 0.000 | 0.000 |
| 23.94° | 0.020 | 0.326 | 0.465 | 0.168 | 0.020 | 0.001 | 0.000 | 0.000 | 0.000 |
| 26.52° | 0.006 | 0.213 | 0.495 | 0.248 | 0.037 | 0.002 | 0.000 | 0.000 | 0.000 |
| 29.34° | 0.001 | 0.113 | 0.464 | 0.349 | 0.070 | 0.004 | 0.000 | 0.000 | 0.000 |
| 31.65° | 0.000 | 0.059 | 0.394 | 0.426 | 0.113 | 0.007 | 0.000 | 0.000 | 0.000 |
| 33.84° | 0.000 | 0.029 | 0.308 | 0.477 | 0.171 | 0.015 | 0.000 | 0.000 | 0.000 |
| 36.49° | 0.000 | 0.011 | 0.204 | 0.494 | 0.260 | 0.031 | 0.001 | 0.000 | 0.000 |
| 39.06° | 0.000 | 0.004 | 0.121 | 0.457 | 0.355 | 0.062 | 0.002 | 0.000 | 0.000 |
| 41.05° | 0.000 | 0.001 | 0.063 | 0.378 | 0.440 | 0.113 | 0.005 | 0.000 | 0.000 |
| 43.74° | 0.000 | 0.000 | 0.035 | 0.299 | 0.483 | 0.172 | 0.011 | 0.000 | 0.000 |
| 46.27° | 0.000 | 0.000 | 0.015 | 0.204 | 0.493 | 0.262 | 0.025 | 0.000 | 0.000 |
| 48.35° | 0.000 | 0.000 | 0.007 | 0.139 | 0.463 | 0.342 | 0.047 | 0.001 | 0.000 |
| 50.94° | 0.000 | 0.000 | 0.003 | 0.079 | 0.389 | 0.432 | 0.095 | 0.002 | 0.000 |
| 53.51° | 0.000 | 0.000 | 0.001 | 0.042 | 0.295 | 0.486 | 0.169 | 0.007 | 0.000 |
| 56.16° | 0.000 | 0.000 | 0.000 | 0.020 | 0.202 | 0.490 | 0.269 | 0.020 | 0.000 |
| 58.35° | 0.000 | 0.000 | 0.000 | 0.011 | 0.137 | 0.451 | 0.358 | 0.043 | 0.000 |
| 60.66° | 0.000 | 0.000 | 0.000 | 0.005 | 0.087 | 0.382 | 0.439 | 0.086 | 0.001 |
| 63.48° | 0.000 | 0.000 | 0.000 | 0.002 | 0.047 | 0.281 | 0.492 | 0.174 | 0.003 |
| 66.06° | 0.000 | 0.000 | 0.000 | 0.001 | 0.026 | 0.196 | 0.482 | 0.284 | 0.012 |
| 68.62° | 0.000 | 0.000 | 0.000 | 0.001 | 0.015 | 0.129 | 0.421 | 0.397 | 0.037 |
| 71.08° | 0.000 | 0.000 | 0.000 | 0.000 | 0.009 | 0.085 | 0.336 | 0.476 | 0.093 |
| 73.06° | 0.000 | 0.000 | 0.000 | 0.000 | 0.007 | 0.061 | 0.266 | 0.495 | 0.170 |
| 75.04° | 0.000 | 0.000 | 0.000 | 0.000 | 0.006 | 0.047 | 0.203 | 0.464 | 0.279 |
| 76.70° | 0.000 | 0.000 | 0.000 | 0.001 | 0.008 | 0.043 | 0.160 | 0.394 | 0.394 |

Table S3 The leaf nitrogen fraction and absolute content in different layers of canopy under 28 vertical leaf nitrogen distributions

| Canopy Layer | Leaf nitrogen content | | | | | | | | | | | | | |
| --- | --- | --- | --- | --- | --- | --- | --- | --- | --- | --- | --- | --- | --- | --- |
|  | Distribution 1  (N1) | | Distribution 2  (N2) | | Distribution 3  (N3) | | Distribution 4  (N4) | | Distribution 5  (N5) | | Distribution 6  (N6) | | Distribution 7  (N7) | |
|  | Fraction | Content  (mg) | Fraction | Content  (mg) | Fraction | Content  (mg) | Fraction | Content  (mg) | Fraction | Content  (mg) | Fraction | Content  (mg) | Fraction | Content  (mg) |
| CL1 | 0.143 | 128 | 0.125 | 113 | 0.108 | 97 | 0.091 | 82 | 0.073 | 66 | 0.063 | 56 | 0.054 | 48 |
| CL2 | 0.178 | 157 | 0.171 | 154 | 0.165 | 149 | 0.157 | 141 | 0.145 | 130 | 0.136 | 122 | 0.126 | 114 |
| CL3 | 0.170 | 153 | 0.173 | 156 | 0.175 | 158 | 0.175 | 158 | 0.173 | 155 | 0.169 | 152 | 0.165 | 149 |
| CL4 | 0.151 | 136 | 0.158 | 142 | 0.163 | 147 | 0.168 | 151 | 0.173 | 155 | 0.174 | 157 | 0.175 | 158 |
| CL5 | 0.126 | 114 | 0.132 | 119 | 0.139 | 125 | 0.146 | 131 | 0.153 | 138 | 0.159 | 143 | 0.163 | 147 |
| CL6 | 0.098 | 89 | 0.103 | 92 | 0.108 | 97 | 0.115 | 103 | 0.123 | 110 | 0.129 | 116 | 0.134 | 121 |
| CL7 | 0.070 | 63 | 0.072 | 65 | 0.075 | 68 | 0.080 | 72 | 0.086 | 78 | 0.092 | 82 | 0.097 | 87 |
| CL8 | 0.043 | 39 | 0.043 | 39 | 0.044 | 40 | 0.047 | 42 | 0.050 | 45 | 0.054 | 48 | 0.058 | 52 |
| CL9 | 0.020 | 18 | 0.019 | 17 | 0.019 | 17 | 0.019 | 17 | 0.021 | 19 | 0.022 | 20 | 0.024 | 21 |
| CL10 | 0.004 | 3 | 0.003 | 3 | 0.003 | 3 | 0.003 | 3 | 0.003 | 3 | 0.003 | 3 | 0.004 | 3 |

Continued

| Canopy Layer | Nitrogen content | | | | | | | | | | | | | |
| --- | --- | --- | --- | --- | --- | --- | --- | --- | --- | --- | --- | --- | --- | --- |
|  | Distribution 8  (N8) | | Distribution 9  (N9) | | Distribution 10  (N10) | | Distribution 11  (N11) | | Distribution 12  (N12) | | Distribution 13  (N13) | | Distribution 14  (N14) | |
|  | Fraction | Content  (mg) | Fraction | Content  (mg) | Fraction | Content  (mg) | Fraction | Content  (mg) | Fraction | Content  (mg) | Fraction | Content  (mg) | Fraction | Content  (mg) |
| CL1 | 0.044 | 40 | 0.037 | 34 | 0.032 | 28 | 0.027 | 24 | 0.023 | 21 | 0.019 | 18 | 0.016 | 14 |
| CL2 | 0.115 | 104 | 0.105 | 95 | 0.097 | 87 | 0.088 | 79 | 0.081 | 73 | 0.073 | 66 | 0.064 | 58 |
| CL3 | 0.159 | 143 | 0.153 | 138 | 0.146 | 132 | 0.139 | 126 | 0.133 | 119 | 0.125 | 113 | 0.116 | 104 |
| CL4 | 0.175 | 158 | 0.174 | 157 | 0.172 | 155 | 0.169 | 152 | 0.166 | 149 | 0.161 | 145 | 0.155 | 139 |
| CL5 | 0.167 | 151 | 0.171 | 154 | 0.173 | 156 | 0.175 | 157 | 0.175 | 158 | 0.175 | 158 | 0.174 | 157 |
| CL6 | 0.141 | 127 | 0.147 | 133 | 0.153 | 137 | 0.158 | 142 | 0.162 | 146 | 0.166 | 149 | 0.170 | 153 |
| CL7 | 0.104 | 94 | 0.111 | 100 | 0.117 | 105 | 0.123 | 111 | 0.129 | 116 | 0.136 | 122 | 0.144 | 130 |
| CL8 | 0.063 | 57 | 0.068 | 61 | 0.073 | 66 | 0.079 | 71 | 0.085 | 77 | 0.092 | 83 | 0.101 | 91 |
| CL9 | 0.026 | 24 | 0.029 | 26 | 0.032 | 29 | 0.036 | 32 | 0.039 | 35 | 0.044 | 40 | 0.051 | 46 |
| CL10 | 0.004 | 4 | 0.004 | 4 | 0.005 | 5 | 0.006 | 5 | 0.007 | 6 | 0.008 | 7 | 0.010 | 9 |

Continued

| Canopy Layer | Nitrogen content | | | | | | | | | | | | | |
| --- | --- | --- | --- | --- | --- | --- | --- | --- | --- | --- | --- | --- | --- | --- |
|  | Distribution 15  (N15) | | Distribution 16  (N16) | | Distribution 17  (N17) | | Distribution 18  (N18) | | Distribution 19  (N19) | | Distribution 20  (N20) | | Distribution 21  (N21) | |
|  | Fraction | Content  (mg) | Fraction | Content  (mg) | Fraction | Content  (mg) | Fraction | Content  (mg) | Fraction | Content  (mg) | Fraction | Content  (mg) | Fraction | Content  (mg) |
| CL1 | 0.013 | 12 | 0.011 | 9 | 0.009 | 8 | 0.008 | 7 | 0.007 | 6 | 0.006 | 6 | 0.005 | 5 |
| CL2 | 0.056 | 51 | 0.049 | 44 | 0.044 | 40 | 0.040 | 36 | 0.036 | 33 | 0.033 | 30 | 0.030 | 27 |
| CL3 | 0.106 | 96 | 0.097 | 88 | 0.090 | 81 | 0.084 | 76 | 0.078 | 71 | 0.073 | 66 | 0.067 | 61 |
| CL4 | 0.148 | 133 | 0.140 | 126 | 0.134 | 120 | 0.128 | 115 | 0.121 | 109 | 0.115 | 104 | 0.108 | 98 |
| CL5 | 0.172 | 155 | 0.168 | 151 | 0.164 | 148 | 0.160 | 144 | 0.156 | 140 | 0.151 | 136 | 0.145 | 130 |
| CL6 | 0.173 | 156 | 0.175 | 157 | 0.175 | 158 | 0.175 | 158 | 0.174 | 157 | 0.172 | 155 | 0.169 | 152 |
| CL7 | 0.152 | 136 | 0.158 | 143 | 0.163 | 147 | 0.167 | 150 | 0.170 | 153 | 0.173 | 156 | 0.175 | 157 |
| CL8 | 0.110 | 99 | 0120 | 108 | 0.128 | 115 | 0.135 | 121 | 0.142 | 128 | 0.149 | 134 | 0.156 | 140 |
| CL9 | 0.058 | 52 | 0.067 | 60 | 0.074 | 67 | 0.081 | 73 | 0.090 | 81 | 0.099 | 89 | 0.109 | 98 |
| CL10 | 0.120 | 11 | 0.015 | 13 | 0.018 | 16 | 0.021 | 19 | 0.025 | 22 | 0.030 | 27 | 0.035 | 32 |

Continued

| Canopy Layer | Nitrogen content | | | | | | | | | | | | | |
| --- | --- | --- | --- | --- | --- | --- | --- | --- | --- | --- | --- | --- | --- | --- |
|  | Distribution 22  (N22) | | Distribution 23  (N23) | | Distribution 24  (N24) | | Distribution 25  (N25) | | Distribution 26  (N26) | | Distribution 27  (N27) | | Distribution 28  (N28) | |
|  | Fraction | Content  (mg) | Fraction | Content  (mg) | Fraction | Content  (mg) | Fraction | Content  (mg) | Fraction | Content  (mg) | Fraction | Content  (mg) | Fraction | Content  (mg) |
| CL1 | 0.005 | 4 | 0.004 | 4 | 0.004 | 4 | 0.004 | 4 | 0.004 | 4 | 0.004 | 4 | 0.005 | 5 |
| CL2 | 0.027 | 24 | 0.025 | 23 | 0.023 | 21 | 0.022 | 20 | 0.021 | 19 | 0.021 | 19 | 0.022 | 20 |
| CL3 | 0.062 | 56 | 0.058 | 52 | 0.054 | 49 | 0.050 | 45 | 0.047 | 43 | 0.046 | 42 | 0.046 | 41 |
| CL4 | 0.101 | 91 | 0.096 | 86 | 0.090 | 81 | 0.084 | 75 | 0.079 | 71 | 0.075 | 68 | 0.073 | 66 |
| CL5 | 0.138 | 124 | 0.132 | 119 | 0.126 | 114 | 0.118 | 106 | 0.112 | 100 | 0.106 | 96 | 0.102 | 91 |
| CL6 | 0.165 | 149 | 0.161 | 145 | 0.156 | 141 | 0.149 | 134 | 0.142 | 128 | 0.136 | 122 | 0.129 | 116 |
| CL7 | 0.175 | 158 | 0.175 | 158 | 0.174 | 156 | 0.170 | 153 | 0.165 | 149 | 0.160 | 144 | 0.154 | 138 |
| CL8 | 0.162 | 146 | 0.167 | 150 | 0.171 | 154 | 0.175 | 157 | 0.175 | 158 | 0.174 | 157 | 0.171 | 154 |
| CL9 | 0.120 | 108 | 0.130 | 117 | 0.139 | 125 | 0.152 | 137 | 0.162 | 146 | 0.169 | 152 | 0.174 | 157 |
| CL10 | 0.043 | 39 | 0.051 | 46 | 0.061 | 55 | 0.076 | 69 | 0.092 | 83 | 0.108 | 97 | 0.124 | 112 |

Fig. S1 Simulated effects of vertical distribution of leaf area and leaf nitrogen on ADM in maize canopy at the LAI of 3 (A), 4 (B), 5 (C), 6 (D), and 7 (E). Normalized ADM, which was normalized by its maximal for maize canopies with different LAI, was denoted by partially filled pie charts with color gradients. Both the filled portion and color gradient indicate the magnitude of normalized ADM. The ADM value was also labelled in the middle of each partially filled pie chart.

Fig. S2 Simulated effects of vertical distribution of leaf area and leaf nitrogen on RUE in maize canopy at the LAI of 3 (A), 4 (B), 5 (C), 6 (D), and 7 (E). Normalized RUE, which was normalized by its maximal for maize canopies with different LAI, was denoted by partially filled pie charts with color gradients. Both the filled portion and color gradient indicate the magnitude of normalized RUE. The RUE value was also labelled in the middle of each partially filled pie chart.
